# Transcriptomic data of pre-meiotic stage of floret development in apomictic and sexual types of guinea grass (*Panicum maximum* Jacq.)

**DOI:** 10.1016/j.dib.2018.03.001

**Published:** 2018-03-10

**Authors:** Auji Radhakrishna, Krishna Kumar Dwivedi, Manoj Kumar Srivastava, A.K. Roy, D.R. Malaviya, P. Kaushal

**Affiliations:** a ICAR-Indian Grassland and Fodder Research Institute, Jhansi 284003, UP, India; b ICAR-Indian Institute of Sugarcane Research, Lucknow 226002, UP, India; c ICAR-National Institute of Biotic Stress Management, Raipur 493225, Chhattishgarh, India

**Keywords:** Florets, Pre-meiosis, Apomixis, RNA-Seq, *Panicum maximum*

## Abstract

Guinea grass (*Panicum maximum* Jacq), an important fodder crop of humid and sub-humid tropical regions, reproduces through apomixis, a method of clonal propagation through seeds. Lack of knowledge of the genetic and molecular control of this phenomena has hindered the genetic improvement of this crop. The dataset provided here represents the first RNA-Seq based assembly and analysis of florets at pre-meiotic stage from the apomictic and sexual genotypes of guinea grass. The raw sequence files in FASTQ format were deposited in the NCBI SRA database with accession number SRP115883. A total of 24.8 Gb raw sequence data, corresponding to 17,96,65,827 raw reads was obtained by paired end sequencing. We used Trinity for de-novo assembly and identified 57,647 transcripts in sexual and 49,093 transcripts in apomictic type. This transcriptome data will be useful for identification and comparative analysis of genes regulating the mode of reproduction in grasses.

**Specifications Table**

**Table:** 

Subject area	Biology
More specific subject area	Genomics and Bionformatics
Type of data	Transcriptome data
How data was acquired	Illumina HiSeq™ 2500
Data format	Raw FASTQ
Experimental factors	Differentially expressed transcripts from apomictic and sexual genotypes
Experimental features	Florets at pre- meiotic stage of development were sampled for transcriptome sequencing and de novo assembled using Trinity.
Data source location	Jhansi, India (78°35′ E 25°26′ N)
Data accessibility	Raw reads from apomictic and sexual genotypes of *P. maximum* were deposited in the NCBI SRA database with accession number SRP115883
	(https://www.ncbi.nlm.nih.gov/sra/?term=SRP115883)

**Value of the data**

• The present study reports the first transcriptome profiling of the reproductive tissues in guinea grass.
• This dataset is valuable for the identification of differentially expressed transcripts during the pre-meiotic stage of floret development in apomictic and sexual genotypes.
• The availability of these datasets will help to gain further insights into the molecular mechanisms regulating apomixis in guinea grass.

## 1 Data

Apomixis in guinea grass is believed to be controlled by many genes and unlikely by a single block [1]. Broadly, the differentiation in reproductive pathway of sexual lines differs from apomictic at three stages of development *viz*., pre-meiotic (programming of ovule to enter into apomeiotic/meiotic pathway), meiotic (cell divisions in ovule to develop unreduced or reduced embryo-sac based on pre-meiotic programming) and post-meiotic (embryo-sac maturation and preparing for embryo development either parthenogenetically or zygotic). Genetic analysis involving lines expressing high frequency of individual components can be useful for better understanding of apomixis and its components in guinea grass.

Transcriptome data reported here was generated from the spikes representing the pre-meiotic development stage of apomictic and sexual genotypes of *P. maximum*. Raw reads obtained from both the apomictic and sexual genotypes of *P. maximum* were deposited in the NCBI SRA database with accession number SRP115883 (https://www.ncbi.nlm.nih.gov/sra/?term=SRP115883).

(https://www.ncbi.nlm.nih.gov/sra/?term=SRP115883). Short reads were filtered, processed, assembled and analyzed as described in the next section. Trinity was used for de-novo assembly which resulted in identification of 57,647 transcripts in sexual and 49,093 transcripts in apomictic type. The transcriptome sequencing and assembly are summarized in Table 1.

**Table 1:** Statistics of sequencing and assembly of *P. maximum* transcriptome.

**Attribute**	**Sexual**	**Apomictic**
Total filtered reads	80,935,053	98,730,774
Alignment Percentage	92.4	96.5
Total assembled contigs	256,390	353,345
Number of transcripts with FPKM >= 1.0	174,772	232,058
Longest transcript length (bp)	16,671	16,933
Mean GC %of transcripts	44.67	45.04
Number of transcripts with significant BLASTX match	112,441 (64.3%)	146,598 (63.1%)
Number of transcripts with UniProt annotation	73,422 (42%)	94,641 (40.8%)
No. of *de novo* transcripts	57,647	49,093

## 2 Experimental design, materials and methods

### 2.1 Plant material and transcriptome sequencing

Spikes from two divergent genotypes of *P. maximum* were sampled for RNA sequencing: the sexual accession SPM92 and an apomictic cultivar BG-1. Individual florets were harvested from two biological replicates and immediately frozen in liquid nitrogen. About 20 florets from the individual plant were pooled and used for total RNA extraction using the Qiagen Plant RNeasy kit protocol (Qiagen, Germany). RNA quality was determined using Agilent Tapestation instrument and RNA screen tape. RIN value of sample was used as indicator for intactness of RNA. For mRNA library preparation a Truseq RNA sample prep kit with plant Ribozero (Ilumina, San Diego, U.S.A.) was used. In brief, Ribo-Zero Plant kit depletes cytoplasmic and chloroplast rRNA, following purification, the RNA is fragmented into small pieces and first strand cDNA is synthesized using reverse transcriptase and random primers, followed by second strand cDNA synthesis using DNA Polymerase I and RNase H. Single 'A' base is added to the cDNA fragments prior to ligation of the adapter. The products are purified and enriched with PCR to create the final cDNA library. The different samples were bar-coded with individual unique indices for multiplexing during sequencing. Paired end RNA Sequencing was carried out by Scigenomics Co (Kochi, India) using Illumina Hiseq. 2500 platform at 2×100 bp in the high throughput mode.

### 2.2 *De novo* assembly and annotation

We obtained a total of 22,68,88,698 paired end reads using Illumina technology, which generated 24.88 GB of data. Raw reads were cleaned by removing illumina adapter sequences using Cutadapt v1.8 [2]. Trimming of poor quality bases (phred score <= 30) using Sickle v1.33 [3]; resulted in 17,96,65,827 reads with an average length of 82 bp. The quality filtered reads were selected for *de novo* assembly using Trinity software [4]; a reference genome-independent assembler which identifies transcripts using three independent modules: Inchworm, Butterfly and Chrysalis. The assembled contigs were used later as a reference transcriptome for the purpose of determining differential gene expression. The filtered reads were aligned to the corresponding contigs using Bowtie2 program [5]; allowing 1 mismatch in the seed region (length = 31 bp). The expression value for all transcripts was calculated by using FPKM method (fragments per kilobase of exon model per million mapped reads), a length normalized measure of relative abundance of transcript that allows expression levels to be compared within or between different samples [6].
