# Supplementary material for: Transcriptomic data of pre-meiotic stage of floret development in apomictic and sexual types of guinea grass (Panicum maximum Jacq.)
Source: Data Brief. 2018 Mar 10;18:590–3. doi: 10.1016/j.dib.2018.03.001 (PMC5995770; doi:10.1016/j.dib.2018.03.001)
Supplement: Supplementary file 1 — Supplementary material [file mmc1.docx]

**AUTHOR DECLARATION**

By submitting this manuscript to journal Data in Brief, we wish to confirm that there are no known conflicts of interest associated with this publication and there has been no significant financial support for this work that could have influenced its outcome.

We confirm that the manuscript has been read and approved by all named authors and that there are no other persons who satisfied the criteria for authorship but are not listed.

We further confirm that the order of authors listed in the manuscript has been approved by all of us.

**Signature:**

Pankaj Kaushal

Corresponding Author (Duly authorized by all contributing authors)

Date: 14 November 2017
